# RcgA and RcgR, Two Novel Proteins Involved in the Conjugative Transfer of Rhizobial Plasmids

**DOI:** 10.1128/mbio.01949-22

**Published:** 2022-09-08

**Authors:** Lucas G. Castellani, Abril Luchetti, Juliet F. Nilsson, Julieta Pérez-Giménez, Ben Struck, Andreas Schlüter, Alfred Pühler, Karsten Niehaus, David Romero, Mariano Pistorio, Gonzalo Torres Tejerizo

**Affiliations:** a Instituto de Biotecnología y Biología Molecular, CCT-La Plata-CONICET, Departamento de Ciencias Biológicas, Facultad de Ciencias Exactas, Universidad Nacional de La Plata, La Plata, Argentina; b Center for Biotechnology (CeBiTec), Bielefeld University, Genome Research of Industrial Microorganisms, Bielefeld, Germany; c Programa de Ingeniería Genómica, Centro de Ciencias Genómicas, Universidad Nacional Autónoma de México, Cuernavaca, Morelos, México; National University of Singapore

**Keywords:** Rhizobia, plasmid, conjugation, Rhizobium, gene regulation

## Abstract

Rhizobia are Gram-negative bacteria that are able to establish a nitrogen-fixing symbiotic interaction with leguminous plants. Rhizobia genomes usually harbor several plasmids which can be transferred to other organisms by conjugation. Two main mechanisms of the regulation of rhizobial plasmid transfer have been described: quorum sensing (QS) and the *rctA*/*rctB* system. Nevertheless, new genes and molecules that modulate conjugative transfer have recently been described, demonstrating that new actors can tightly regulate the process. In this work, by means of bioinformatics tools and molecular biology approaches, two hypothetical genes are identified as playing key roles in conjugative transfer. These genes are located between conjugative genes of plasmid pRfaLPU83a from Rhizobium favelukesii LPU83, a plasmid that shows a conjugative transfer behavior depending on the genomic background. One of the two mentioned genes, *rcgA*, is essential for conjugation, while the other, *rcgR*, acts as an inhibitor of the process. In addition to introducing this new regulatory system, we show evidence of the functions of these genes in different genomic backgrounds and confirm that homologous proteins from non-closely related organisms have the same functions. These findings set up the basis for a new regulatory circuit of the conjugative transfer of plasmids.

## INTRODUCTION

Plasmids are extrachromosomal elements that encode different genes and can be horizontally transferred between organisms. Their ability to be transferred to other microorganisms via conjugation makes plasmids one of the most important vehicles for bacterial evolution, since the acquisition of a plasmid confers new phenotypic traits to the receptor strain (1–3). Among the functions that plasmids can encode, antibiotic resistance genes and determinants of pathogenicity or symbiosis are of great relevance to bacterial adaptation to novel ecological niches (4, 5).

Plasmids are widely distributed among soil-living microorganisms, such as Rhizobia. Rhizobia are a group of bacteria that are able to inhabit soil as free-living microorganisms or in symbiosis with leguminous plants (6, 7). Rhizobia can harbor different numbers of plasmids, ranging in size between 10 Kb and 1.5 Mb. Based on the functions that plasmids encode, they can be classified into symbiotic plasmids (pSyms), which harbor a set of genes that enables a complete symbiosis; or into cryptic or accessory plasmids, which encode functions that are not related to the symbiosis (8, 9). Accessory plasmids can be important for the adaptation of rhizobia to different environments, and they can also affect the symbiotic process (10–13). Attention has been paid to the conjugative transfer (CT) of rhizobial plasmids, mainly due to the consequences that pSyms and accessory plasmids can have on symbiotic relationships (14, 15). Studies on rhizobial CT have led to the discovery and understanding of novel mechanisms of CT regulation (16, 17).

Two main molecular mechanisms of rhizobial plasmid transfer regulation have been described. Some plasmids, such as pRetCFN42d from Rhizobium etli CFN42, are regulated by the genes *rctA/rctB*. Briefly, *rctA* constitutively represses conjugation (in laboratory conditions), and, after an unknown signal, *rctB*, which encodes an RctA inhibitory protein, is expressed, thereby allowing the expression of CT genes (18–20). Other plasmids, such as pTiC58 from Agrobacterium tumefaciens C58, are regulated by a quorum sensing (QS) mechanism, which is based on the sensing of diffusible molecules (21, 22). In most cases, this molecule is an acyl-homoserine-lactone (AHL), which is synthesized by TraI, the AHL synthase, which is generally localized in the conjugative region of the regulated plasmid (23). AHLs bind to the transcriptional regulator TraR, thereby allowing the expression of conjugative genes (22, 24). Rhizobial plasmids whose conjugative regulation is mediated by TraR have been classified into four groups based on a phylogenetic analysis of TraR (25). Also, similarities between the organizations of the CT genes of plasmids from each group were observed. Plasmid pRfaLPU83a (here, pLPU83a), an accessory plasmid from Rhizobium favelukesii LPU83 (here, LPU83), encodes a *traR* gene, but it lacks a *traI* gene in the conjugative region. Along with plasmids with the mentioned genetic organization and TraR phylogenetic analysis results, pLPU83a belongs to group I-C. Interestingly, it has been demonstrated that the CT of pLPU83a does not respond to AHLs or to a QS mechanism (25). Nevertheless, TraR is essential for plasmid transfer (26). Another striking feature of pLPU83a is that it is capable of changing its conjugative behavior, depending on the genomic background. This plasmid is able to conjugate from the parental strain and from some hosts, but not from others (27), indicating some differences in the regulatory network of each background. Altogether, the previous evidence suggests that a different regulatory system is involved in CT.

Progress in DNA sequencing technologies has made it possible to obtain thousands of genome sequences in recent years. As a consequence, the presence of many genes with no described functions (hypothetical genes) has been observed. In particular, the study of hypothetical genes encoded in rhizobial plasmids has allowed for the identification of several genes involved in the conjugative process. Ding et al. (28) described a gene encoding a transcriptional regulator that is involved in the transfer regulation of plasmid pRleVF39b from Rhizobium leguminosarum VF39. This gene, called *trbR*, is located between the DNA transfer and replication (Dtr) and mating pair formation (Mpf) regions of pRleVF39b. In the same genetic region, Wathugala et al. (29) reported the presence of six more hypothetical genes that are also involved in CT. Lopez-Fuentes et al. (30) reported three hypothetical genes located between the Dtr and Mpf regions that were involved in the CT of pRetCFN42a from Rhizobium etli CFN42, whose regulation is mediated by QS. We found two conserved hypothetical genes in all of the plasmids belonging to group I-C, located in tandem between the Dtr and Mpf regions (25). Considering that the presence of hypothetical genes located between the Dtr and Mpf regions could be related to the CT mechanism and that the position of these genes in the plasmids of group I-C is conserved, a role in CT could be inferred. In this work, we studied three hypothetical genes and their distribution in the environment using approaches from bioinformatics, and we then confirmed their association with the CT of plasmid pLPU83a. Also, methods to obtain a first insight into this new molecular mechanism were performed. Finally, we analyzed the roles of similar proteins from other organisms, which revealed that they play a similar role, suggesting a conserved function for these proteins.

## RESULTS

### *In silico* characterization and taxonomic distribution of LPU83a_0145, LPU83a_0146, and LPU83a_0148.

Two hypothetical genes were found in tandem between the Dtr and Mpf regions in all of the plasmids belonging to rhizobial group I-C (25). In the plasmid pLPU83a from LPU83, there is a third hypothetical gene in tandem, located downstream and in the same orientation as the other two, with the difference being that this gene is not conserved in all of the plasmids of group I-C. In pLPU83a, these genes are LPU83a_0145, LPU83a_0146 (conserved), and LPU83a_0148 (conserved). By means of BLAST and Pfam, only one domain corresponding to a putative α/β hydrolase was identified for LPU83a_0146 (Fig. S1A, available at http://sedici.unlp.edu.ar/handle/10915/140513). Proteins similar to LPU83a_0148 showed a transmembrane protein annotation. Thus, TMHMM-2.0 (31, 32), a software for the prediction of transmembrane domains, was used to predict those domains. The results showed six transmembrane domains in LPU83a_0148 (Fig. S1B). LPU83a_0145 did not show any described domain. All three proteins were modeled with Alphafold (33). Despite the fact that the three-dimensional (3-D) structures obtained in this way are prediction models, these structures were compared against the Protein Data Bank (PDB) database, using the Dali server (34) to find structural similarities. LPU83a_0145 showed alpha helix and beta sheet domains, but the structural comparison scores with other proteins were rather low. Thus, no inferences could be made (Fig. S2A, available at http://sedici.unlp.edu.ar/handle/10915/140513). LPU83a_0146 also presented alpha helix and beta sheet domains (Fig. S2B), and it showed structural similarities to acetylxylanesterases. For LPU83a_0148, α-helix domains were predicted, and they would make it possible for this protein to anchor to the membrane. These domains showed similarity to insect olfactory receptors. The portion corresponding to the C terminus of LPU83a_0148 was modeled as a different domain (outside the α-helix domains), and it showed structural similarities to HTH domains, which could be related to transcriptional regulators (Fig. S2C).

In order to know if the hypothetical proteins were present in other organisms, we searched on the NCBI nr database for proteins with more than 30% identity with LPU83a_0145, LPU83a_0146, and LPU83a_0148, with at least 70% coverage of each protein. For LPU83a_0145, we found 1,142 homologous proteins, of which 47.55% corresponded to Proteobacteria (Fig. S3A, available at http://sedici.unlp.edu.ar/handle/10915/140513). Within this phylum, most of the organisms belonged to γ-proteobacteria (16.99% of the total hits) and α-proteobacteria (16.55% of the total hits). For LPU83a_0146, we found 1,402 homologous proteins, of which 54.92% were encoded by Proteobacteria and 41.51% by Acidobacteria (Fig. S3B). Within Proteobacteria, almost all of the microorganisms belonged to α-proteobacteria (54.42% of the total hits), with Rhizobiales being the predominant order (48.28% of the total hits). In the case of LPU83a_0148, we found 786 homologous proteins, all of them belonging to Proteobacteria (Fig. S3C). For this protein, 99.49% of the total hits were found in α-proteobacteria. Within this class, 79.39% of the total hits belonged to Rhizobiales while 19.08% belonged to Rhodobacterales. It is also noteworthy that, in most cases, homologs to LPU83a_0146 were found downstream of LPU83a_0148 homologs. Moreover, the analysis of the genetic regions where homologous genes to LPU83a_0146 and LPU83a_0148 are present showed that determinants involved in conjugation are encoded among neighboring genes, allowing us to presume a role for these genes in CT. We also searched these proteins in the metagenomics proteins database. Interestingly, we found hits in the metagenomes of marine sediments, freshwater, and mine drainage, among others (Table S1, available at http://sedici.unlp.edu.ar/handle/10915/140513), extending the environments in which functions of these genes could be relevant.

### Phylogenetic analysis of LPU83a_0146 and LPU83a_0148 homologs.

**FIG 1:**
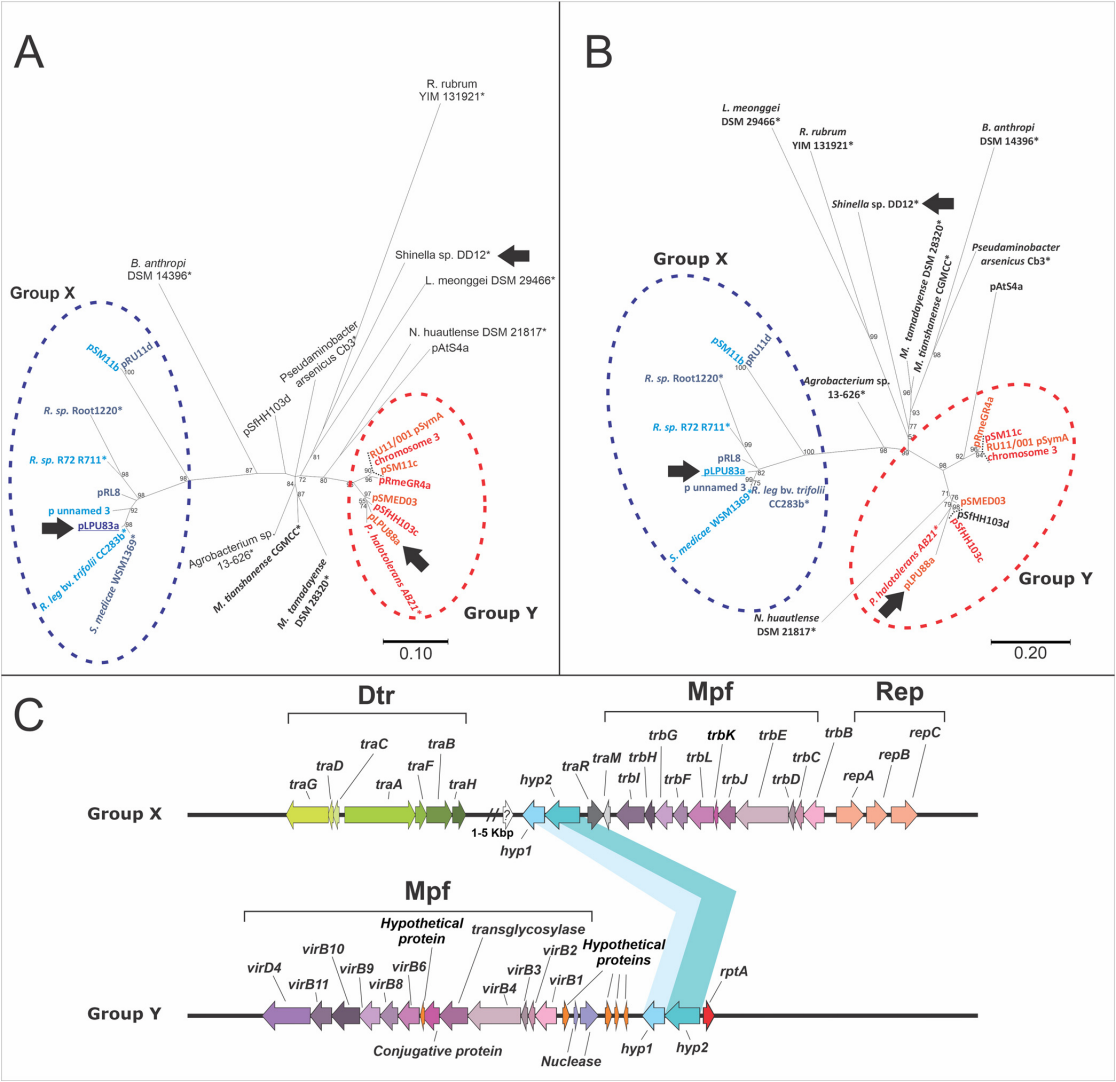
Phylogenetic analysis of LPU83a_0146 and LPU83a_0148. (A) Phylogenetic tree based on LPU83a_0146. (B) Phylogenetic tree based on LPU83a_0148. (C) Genomic organization of conjugative genes in plasmids from groups X and Y. In pLPU83a, genes *hyp1* and *hyp2* correspond to LPU83a_0146 and LPU83a_0148, respectively. The accession numbers of each protein are listed in Table S2. Bootstrap values higher than 50 are shown on the branches. Proteins encoded in plasmids are annotated as the name of the plasmid. Black arrows highlight the hypothetical proteins of pLPU83a and the homologs found in pLPU88a and *Shinella* sp. DD12. The * symbol indicates that a sequence is annotated as a scaffold, meaning that it is not possible to determine whether it belongs to a plasmid.

Since many of the homologous proteins to LPU83a_0146 and LPU83a_0148 were found in tandem, we performed a phylogenetic analysis for homologs to LPU83a_0146 and LPU83a_0148 in order to evaluate whether both proteins showed a similar evolutionary history. For this analysis, we selected organisms with homologs to both proteins which displayed different percentages of identity. We also included several proteins belonging to plasmids found in group I-C (25). The results showed a similar topology between the phylogenetic trees corresponding to the LPU83a_0146 homologs (Fig. 1A) and LPU83a_0148 (Fig. 1B). In both trees, it was possible to observe two well-defined groups of proteins. One of them, group X, contains proteins from pLPU83a and proteins corresponding to plasmids belonging to group I-C. The other cluster, group Y, contains proteins encoded by plasmids corresponding to *Sinorhizobium* strains.

We analyzed the genetic neighborhoods of the analyzed genes in each group. Remarkably, all of them showed genes that were involved in CT. Nevertheless, the conjugative systems present in each group were different. The organization of the conjugative regions observed for the plasmids of group X is the same as that described by Castellani et al. (25) for the plasmids of group I-C. To point it out, *traR* is always located upstream and in the opposite direction of the LPU83a_0148 homologs. The *trb* operon, corresponding to the Mpf, is always located downstream of *traR* (Fig. 1C). In the plasmids of group Y, the Mpf is located downstream of the hypothetical genes, and it is composed by genes similar to those of A. tumefaciens (*virB* genes) (Fig. 1C). Another difference regarding group X is that the coupling protein of the plasmids of group Y (*virD4*) are associated with the Mpf, while in the plasmids of group X (*traG*), it is located within the Dtr region. In addition, Dtr genes and *traR* are absent from the analyzed region of the group Y members. Moreover, the *rptA* gene, whose product participates in CT via an unknown mechanism (35), is located upstream of the LPU83a_0148 homologs in group Y. This analysis allowed us to identify two groups among the plasmids that harbor homologs to LPU83a_0146 and LPU83a_0148.

### Genetic organization of LPU83a_0145, LPU83a_0146, and LPU83a_0148.

The conserved, in tandem organization of LPU83a_0146 and LPU83a_0148 could suggest that both of the genes are expressed as an operon. LPU83a_0145, the third gene located downstream of LPU83a_0146, in the same orientation, could be another element of the same transcriptional unit. A preliminary analysis, using data from a RNA-seq experiment (36), suggested that LPU83a_0146 and LPU83a_0148 are transcribed together and that LPU83a_0145 is transcribed independently (GEO accession number GSE141742). To confirm this organization, we designed specific primers to amplify fragments containing regions of contiguous genes, using cDNA as the template. Thereby, it would be possible to obtain polymerase chain reaction (PCR) fragments only if both contiguous genes were transcribed in the same mRNA. The results showed that LPU83a_0146 and LPU83a_0148 are transcribed as an operon, while LPU83a_0145 is transcribed independently (Fig. S4, available at http://sedici.unlp.edu.ar/handle/10915/140513).

### Functional analysis of LPU83a_0145, LPU83a_0146, and LPU83a_0148.

**FIG 2:**
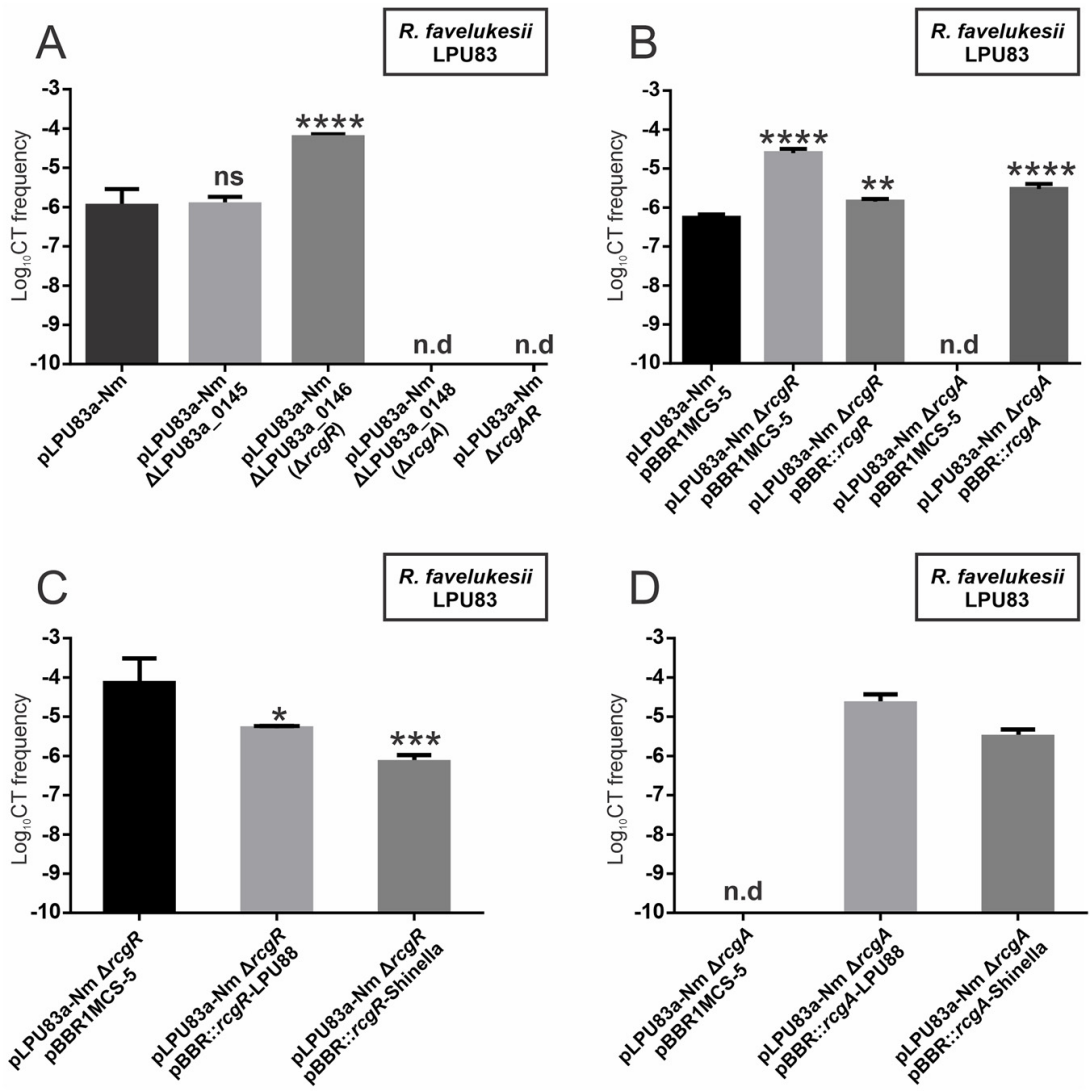
Conjugative transfer frequency of mutants in LPU83a_0145, LPU83a_0146, LPU83a_0148, and the complemented strains. (A) Evaluation of CT frequencies of plasmid pLPU83a-13 and the LPU83a_0145, LPU83a_0146, and LPU83a_0148 mutants of this plasmid. (B) Evaluation of the CT frequencies of plasmid pLPU83a-13 and the LPU83a_0146 and LPU83a_0148 mutants containing the empty vector pBBR1MCS-5 or a vector carrying an entire copy of the deleted gene. (C) CT frequencies of the *rcgR* mutant in the presence of homologous genes encoded by S. meliloti LPU88 and *Shinella* sp. DD12. (D) CT frequencies of the *rcgA* mutant in the presence of homologous genes encoded by S. meliloti LPU88 and *Shinella* sp. DD12. n.d: not detected. A statistical comparison was performed via Dunnett’s multiple comparison test in relation to the wild-type values. ns: not significant. *, *P* = 0.0134; **, *P* = 0.0024; ***, *P* = 0.0004; ****, *P* < 0.0001.

Separate in-frame deletions of the LPU83a_0145, LPU83a_0146, and LPU83a_0148 genes were generated by double crossing-over. Once the deletional mutants were obtained, the CT frequency of each plasmid was evaluated. The LPU83a_0145 mutant showed similar CT frequencies to the wild-type plasmid (Fig. 2A). Deletion in LPU83a_0146 showed a remarkable increase in CT frequencies in comparison with the wild-type values (6.05 ± 1.24 × 10^−5^ versus 1.55 ± 1.28 × 10^−6^) while the mutant in LPU83a_0148 totally abolished the CT of pLPU83a. A deletion mutant of both LPU83a_0146 and LPU83a_0148 was also unable to perform CT.

To corroborate the CT phenotype observed for LPU83a_0146 and LPU83a_0148, we complemented these mutants with a complete wild-type copy of the deleted gene expressed in *trans* from a replicative vector (pBBR1MCS-5). For both mutants, the presence of the wild-type gene allowed CT frequencies similar to those of the wild-type plasmid (Fig. 2B), confirming that the observed phenotypes were due to the absence of each gene.

Due to the observed phenotype of each gene, we decided to name the operon *rcg* (for Rhizobial Conjugative Gene). Specifically, the gene whose product is completely necessary for conjugation (LPU83a_0148) is called *rcgA*, and the gene which reduces CT (LPU83a_0146) is called *rcgR*.

To evaluate whether the functions of *rcgA* and *rcgR* were conserved in other bacteria, heterologous complementation assays were done. For this, two representative strains carrying plasmids with homologous genes were selected: Sinorhizobium meliloti LPU88 (corresponding to group Y) and *Shinella* sp. DD12 (outside groups X and Y; both indicated with arrows in Fig. 1A). The homologous proteins encoded by S. meliloti LPU88 have 66.79% identity with RcgA and 73.8% identity with RcgR, while the proteins encoded by *Shinella* sp. DD12 have 56.99% and 64.76% identity, respectively. The results showed that the homologous genes encoded by S. meliloti LPU88 and *Shinella* sp. DD12 were able to restore the conjugative behavior of pLPU83a in the absence of *rcgR* (Fig. 2C) or *rcgA* (Fig. 2D), confirming the same biological activity in those organisms.

### Functional analysis of *rcgA* and *rcgR* from different genomic backgrounds.

**FIG 3:**
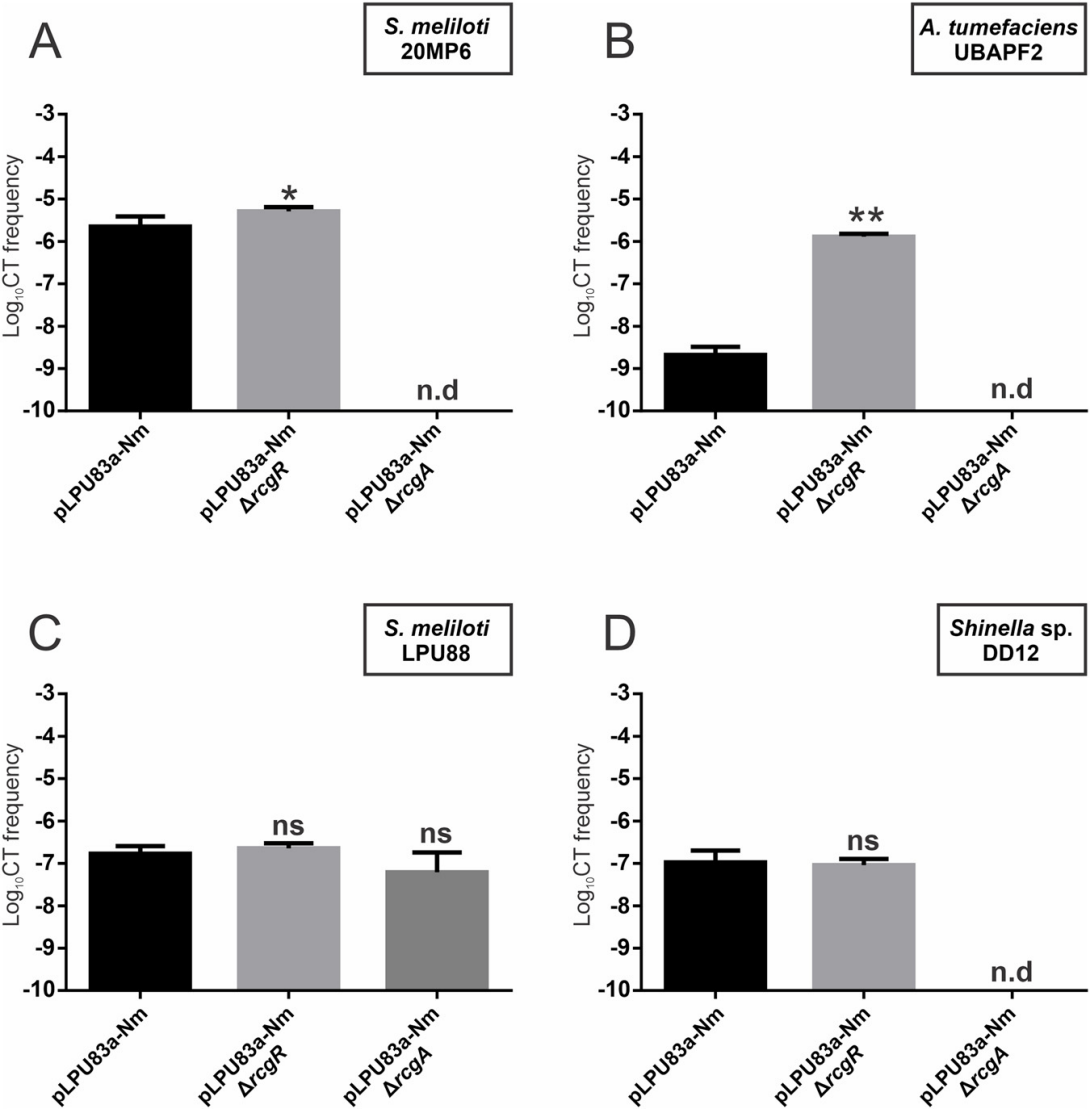
Conjugative transfer frequencies of the plasmid pLPU83a and the *rcgR* and *rcgA* derivative mutants from different genomic backgrounds. (A) CT frequencies from the S. meliloti 20MP6 genomic background. (B) CT frequencies from the A. tumefaciens UBAPF2 genomic background. (C) CT frequencies from the S. meliloti LPU88 genomic background. (D) CT frequencies from the *Shinella* sp. DD12 genomic background. n.d: not detected. A statistical comparison was performed via Dunnett’s multiple comparison test in relation to the wild-type values. ns: not significant. *, *P* = 0.0435; **, *P* < 0.0001.

Plasmid pLPU83a has different CT behaviors depending on the genomic background in which it is located (27). Considering the above, we evaluated the transfer of pLPU83aΔ*rcgA* and pLPU83aΔ*rcgR* from Sinorhizobium meliloti 20MP6 (Fig. 3A) and A. tumefaciens UBAPF2 (here, AtUBAPF2) (Fig. 3B). The transfer of pLPU83a from S. meliloti 20MP6 showed values that were close to those observed from the LPU83 background (2.5 ± 1.5 × 10^−6^ and 1.55 ± 1.28 × 10^−6^, respectively). Plasmids with deletion on *rcgA* were unable to be transferred, and plasmids with deletion on *rcgR* showed a small but statistically significant increase in CT frequency (5.2 ± 1.1 × 10^−6^). When pLPU83a was transferred from AtUBAPF2, we observed CT with a frequency of 2.9 ± 0.86 × 10^−9^, which is near the detection limit. As we expected, it was not possible to obtain the transconjugants of pLPU83aΔ*rcgA*, but pLPU83aΔ*rcgR* showed a remarkable increase in CT frequency compared with the wild-type plasmid (1.3 ± 0.18 × 10^−6^). Thus, *rcgA* seems to be completely necessary for the transfer of pLPU83a from different backgrounds, while *rcgR* acts as a conjugative repressor. In particular, the presence of *rcgR* in AtUBAPF2 represses conjugation in such a way that the transfer is almost undetectable.

Considering that the functions of *rcgA* and *rcgR* are conserved in the transfer of pLPU83a from different backgrounds and that homologous genes from other bacteria are able to complement mutants in the mentioned genes, we evaluated the behaviors of the plasmid and their derivative mutants in the genetic background from which the homologous genes were selected (S. meliloti LPU88 and *Shinella* sp. DD12). The transfer of pLPU83a from S. meliloti LPU88 was carried out with a relatively low frequency in comparison with the transfer from the native background (1.8 ± 0.72 × 10^−7^ and 1.55 ± 1.28 × 10^−6^, respectively). The transfer of pLPU83aΔ*rcgR* showed similar values to those of the wild-type plasmid. For pLPU83aΔ*rcgA*, it was possible to obtain the transconjugants from S. meliloti LPU88 with a frequency of 9.4 ± 0.57 × 10^−7^ (Fig. 3C).

Conjugation of the wild-type plasmid from *Shinella* sp. DD12 was possible at low frequencies, showing no significant differences with the conjugative frequencies of pLPU83aΔ*rcgR* (2.1 ± 1.85 × 10^−08^ and 1.6 ± 1.33 × 10^−08^, respectively). Nevertheless, despite the fact that the homolog to *rcgA* encoded in pDD12 showed the ability to complement our mutant, the transfer of pLPU83aΔ*rcgA* was not observed from this background (Fig. 3D).

### Shotgun proteomics of pLPU83aΔ*rcgA and* pLPU83aΔ*rcgR*.

To further advance the characterization of *rcgA* and *rcgR*, based on the hypothesis that differences in CT frequencies could be due to changes in protein expression, a shotgun proteomic analysis of each mutant and the wild-type strain was performed. Shotgun proteomics allow for the identification of proteins based on the relative abundance of peptides. Three biological replicas of the wild-type, pLPU83aΔ*rcgA*, and pLPU83aΔ*rcgR* were analyzed and quantified through a Label Free Quantification (LFQ) method. Looking only for proteins with significant LFQ intensity values, we were able to identify a total of 1,638 proteins. Among them, 1,202, 1,186 and 1,250 proteins were found in the wild-type strain, pLPU83aΔ*rcgA*, and pLPU83aΔ*rcgR*, respectively (Table S3, available at http://sedici.unlp.edu.ar/handle/10915/140513). Among the identified proteins, the abundance of proteins involved in the Dtr and Mpf of pLPU83a was quite low. The statistical analyses did not reveal proteins with different relative abundances in the comparisons between the wild-type and each mutant. Then, we analyzed the proteins that were detected in one sample but not in the other one, the ON/OFF proteins. When comparing the wild-type strain versus pLPU83aΔ*rcgA*, only 7 proteins were found exclusively in the wild-type strain, while 9 proteins were found exclusively in the pLPU83aΔ*rcgA* strain (Tables S3, red lines). The comparison between the wild-type strain and pLPU83aΔ*rcgR*, showed 3 proteins present exclusively in the wild-type strain, while 3 proteins were exclusive to pLPU83aΔ*rcgR* (Tables S3, yellow lines). Remarkably, two of the proteins present exclusively in pLPU83aΔ*rcgR* were TraR and TraC, the CT master regulator and a protein that is part of the Dtr, respectively. The remaining proteins showed no predicted relationship with CT, nor were they in the plasmid pLPU83a.

### TraR expression analysis using transcriptional fusion.

**FIG 4:**
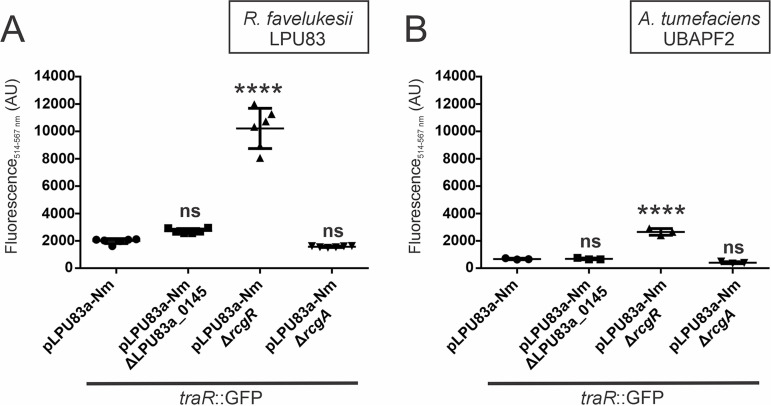
Evaluation of *traR* transcription through fluorescence quantification. (A) Evaluation of the *traR* transcription from the *R. favelukesii* LPU83 genomic background. (B) Evaluation of the *traR* transcription from the A. tumefaciens UBAPF2 genomic background. Each strain carries a GFP protein encoded downstream of *traR*, so the GFP transcription depends on the *traR* promoter activity. A statistical comparison was performed via Dunnett’s multiple comparison test in relation to the wild-type values. ns: not significant. ****, *P*  < 0.0001.

In plasmids whose transfer regulation depends on TraR, the expression of this protein is directly related with transfer frequencies. In these systems, TraR promotes the transcription of the Dtr and Mpf genes (21, 22). As TraR was found in the proteomic analyses, we hypothesized that the phenotype generated by the deletion of each of the studied genes could be related to changes in TraR expression. To evaluate this hypothesis, we constructed a transcriptional fusion between *traR* and a *gfp* gene, and we analyzed it in our mutants. The construction was integrated into pLPU83a derivatives in such a way that *traR* remained functional and that the expression of both genes (*traR* and *gfp*) relied on *traR* promoter. For the strain pLPU83aΔ*rcgA*, no changes in *traR* transcript levels were observed (Fig. 4A), showing that the function of RcgA is not related to the levels of *traR*. For the strain pLPU83aΔ*rcgR*, we observed a significant increase in fluorescence levels (Fig. 4A), demonstrating that RcgR acts by repressing, in some way, *traR* transcription. We also evaluated the plasmid with a deletion in LPU83a_0145, but it did not show changes in *traR* expression.

Moreover, the behaviors of pLPU83a and its mutants were evaluated in the AtUBAPF2 background. The plasmids with the transcriptional fusion were introduced into the mentioned strain, and the fluorescence levels were evaluated. As Fig. 4B shows, the fluorescence values were lower than those obtained from the LPU83 background. Nevertheless, the mutant in *rcgR* showed a significant increase in fluorescence levels, which correlates with the increase in CT frequencies.

## DISCUSSION

Unraveling the molecular basis of plasmid transfer regulation provides an understanding of the behaviors of these molecules in their environments. Molecular mechanisms that regulate CT are diverse; the study of the regulatory networks related to this process aims to understand the dynamics of plasmid DNA dissemination. The plasmid transfer of antibiotic resistance genes has a notorious relevance due to its impact on human health (37, 38). Symbiotic and pathogenic traits are also located in plasmids. In symbiotic bacteria, such as rhizobia, two main mechanisms that regulate CT have been described (18, 19, 21–23). The characterization of hypothetical genes by biochemical and molecular biology approaches has allowed for the identification of new actors involved in the described mechanisms (28–30, 39). In this work, we evaluated the function of three hypothetical genes, and presented evidence of two novel proteins involved in rhizobial plasmid transfer. One of the hypothetical genes, which we named *rcgA*, was predicted as a hypothetical protein with transmembrane domains by approaches from bioinformatics. Modeling with Alphafold (33) confirmed this prediction and showed also a putative HTH motif that is similar to those present in transcriptional regulators. Analyses showed that it is present almost exclusively in Alphaproteobacteria. The other hypothetical gene, *rcgR*, was predicted as a hypothetical protein with an α/β hydrolase domain. The model of this protein showed similarities to esterases. *rcgR* is mostly found in Alphaproteobacteria, but it is also present in Acidobacteria. The predominant distribution of both genes within the Rhizobiales order allows us to suppose that they have a specific function in rhizobia, which has not been extended to other organisms. The conserved organization and the closeness to conjugative genes could suggest a relationship between the functions of both genes and the conjugative process. It is also noteworthy that the environmental distribution is diverse, with the genes being detected in soils, the guts of aquatic organisms, and other environmental metagenomes.

Phylogenetic studies of RcgA and RcgR showed a similar evolutionary history for both proteins, with the obtained trees presenting a matching topology. Two well-defined groups of homologs were observed, with one of them containing proteins close to those encoded by pLPU83a (group X), and the other containing proteins from S. meliloti strains (group Y). It should be noted that group X contains the same plasmids that are present in group I-C, obtained from the TraR homologs (25) and showing an evolutionary relationship, and probably a related function, between the hypothetical genes and *traR*. Despite the plasmids from group Y lacking a *traR* gene, they harbor a complete Mpf set of genes and a coupling protein. Thus, they could be transferred, but the regulation of the CT might be different. Future studies on this system are important, considering that symbiotic plasmids (such as pSymA from Sinorhizobium meliloti RU11/001 and pSM11c from Sinorhizobium meliloti SM11) were found in this group. Until now, most of the described symbiotic plasmids are regulated by *rctA*/*rctB* (18–20), although a QS mechanism has also been described for a symbiotic plasmid from Rhizobium leguminosarum bv. *viciae* (40).

Regarding the biological functions of RcgA and RcgR, we confirmed their participation in the CT of the plasmid pLPU83a. Surprisingly, despite *rcgA* and *rcgR* being transcribed in an operon, they showed opposite effects on conjugation. The deletion of *rcgA* led to a nonconjugative phenotype, while the deletion of *rcgR* showed an increase in CT frequencies. Thus, *rcgA* encodes a protein that is essential for CT, while *rcgR* encodes a protein that, in some way, represses the process. The double mutant, Δ*rcgAR*, did not allow conjugation, indicating that *rcgA* is completely necessary for CT, even in the absence of the repressor. We also confirmed that the biological functions of both proteins are conserved in different organisms, as homologous proteins encoded in plasmids with a different evolutionary relationship and organization of conjugative genes were able to restore the CT phenotypes in complementation assays. Investigating the function of *rcgA* and *rcgR* homologs in the conjugation of plasmids without *traR* will be important in elucidating new regulatory networks.

From an ecological perspective, pLPU83a shows an interesting profile. It is able to use its own conjugative machinery or hijack the machinery of its host (26). Studies of pLPU83a and their derivative mutants in different genomic backgrounds confirmed that *rcgA* and *rcgR* are involved in the CT of pLPU83a when this plasmid is transferred from AtUBAPF2 and S. meliloti 20MP6. The transfer from AtUBAPF2 showed that the genomic background plays an important role in the CT of pLPU83a, as in this background, the wild-type plasmid is transferred at low frequencies, whereas the deletion of *rcgR* notably increases these values. A possible explanation is that in the AtUBAPF2 genomic background, the operon *rcgA*/*rcgR* has an enhanced transcription, resulting in a greater repression of pLPU83a transfer. Another possibility is a reduced transcription of the CT master regulation gene, *traR*, in this background. Experiments using a transcriptional fusion between *traR* and GFP in AtUBAPF2 confirmed that the increments in the CT frequencies of pLPU83aΔ*rcgR* are caused by an enhanced expression of TraR in comparison with the wild-type values. By analyzing the behavior of pLPU83a from other genomic backgrounds, we showed that this plasmid is able to conjugate from S. meliloti LPU88 and *Shinella* sp. DD12. Both strains contain homologous genes to *rcgA* and *rcgR*, and according to heterologous complementation assays, genes of both strains were able to complement the absence of *rcgA* and *rcgR* in the corresponding mutants of pLPU83a in the LPU83 genomic background. Despite the results of the heterologous complementation, it was not possible to obtain the transconjugants of pLPU83aΔ*rcgA* from *Shinella* sp. DD12. A possible explanation for this could be that the homolog to *rcgA* encoded by *Shinella* sp. DD12 is not expressed properly. The fact that there are no differences in CT between the wild-type and Δ*rcgR* plasmid in the S. meliloti LPU88 and *Shinella* sp. DD12 backgrounds could be explained by the *rcgR* homologs encoded in both organisms being functional and repressing CT to similar levels. This could also be supported with the lower CT frequencies observed for pLPU83a in these backgrounds.

Even though the shotgun proteomics assays show a low relative abundance of the proteins involved in CT, it is remarkable that TraR (the master regulator) and TraC (a protein member of the Dtr) were only detectable in pLPU83aΔ*rcgR*. It has been described that, usually, TraR directly activates the transcription of the Dtr and Mpf genes (22, 41) and that, in some systems, TraR is involved in a positive feedback loop (22, 23). In this way, TraR affects different nodes. Our proteomics results, together with the transcriptional fusion assays, allow us to affirm that RcgR is acting as a repressor, somehow affecting the production of TraR and TraC, thereby affecting CT. An interaction between RcgR and TraR (either direct or mediated by another protein) could be affecting the capacity of TraR to binding to its own promoter, thereby affecting the positive feedback loop, and to Dtr and Mpf promoters, thereby affecting the transcription of conjugative genes. In other rhizobial plasmids, a similar mechanism was described for the antiactivator TraM, which affects TraR function by impeding TraR dimerization, a necessary step for TraR function (42–44). A deletion mutant of *traM* in pLPU83a does not show any difference in CT frequencies (not shown). TraM and RcgR do not share any identity by BLASTP. The other regulatory mechanisms of conjugation are based on transcriptional interference in which there is competition between a repressor protein and a transcription factor for binding to the promoter (18–20). Even though there is no predicted DNA interaction domain in RcgR, the protein could be interacting with the *traR* promoter zone, thereby preventing the transcription of the gene.

The overexpression of TraC in the absence of RcgR could reflect an enhancement (but not one that is detectable in our proteomics approaches) in the levels of the proteins belonging to Dtr and Mpf. Thus, an interaction between RcgR and the promoters of Dtr and Mpf genes cannot be disregarded. TraR is able to induce Dtr and Mpf transcription by binding to a consensus sequence located in each promoter zone, called *tra* box (45). pLPU83a has *tra* boxes upstream of its Dtr and Mpf genes (26). Thus, this conserved sequences could be the target for RcgR action.

In QS-regulated systems, the mechanism for TraR activity implies the recognition of a signal molecule that is necessary for the dimerization and activity of the protein (21, 46). In most cases, the recognized molecules are AHLs, which have the same homoserine lactone ring but differ in length and in the structure of the acyl chain (47). Some organisms are able to prevent the accumulation of AHLs by hydrolyzing the homoserine lactone rings of these molecules. For example, Agrobacterium tumefaciens A6 encodes an AHL-lactonase, *attM*, that is involved in the hydrolysis of 3OC8AHL, the molecule that mediates the QS response in this strain (48). Based on the predicted α/β hydrolase domain present in RcgR, this protein could be hydrolyzing a signal molecule that is hypothetically involved in TraR activation (either produced by the cell or imported through RcgA). Thus, the lack of this molecule would not allow the formation of the active form of TraR, and in consequence, the Dtr and Mpf genes would not be activated. With the same hypothesis that RcgR acts to hydrolyze a molecule, another possibility is that it participates in the processing of an unknown molecule, thereby generating a smaller version that abolishes TraR activation. This kind of small inhibitor resembles the inhibitor peptide I from Enterococcus faecalis pCF10, which represses CT (49, 50).

The role of RcgA in CT is more difficult to decipher, as the mutant strain did not show any differences in fluorescence values or in the detected proteins. Although there is a high degree of conservation across type I rhizobial transfer systems that work well without RcgA, and although the proteins that form the Mpf have been widely described (51–54), the presence of predicted transmembrane domains in RcgA allow us to hypothesize a structural role for this protein in group I-C of rhizobial plasmids, with it being an accessory protein of the Mpf or being necessary for Mpf assembly. Thus, the lack of RcgA would not allow Mpf assembly and would thereby prevent CT. Coupling proteins consist of a transmembrane and an ATPase domain, and they are essential for CT due to their role in the recruitment of DNA to the Type IV Secretion System (T4SS) (55, 56). Based on this, despite pLPU83a encoding a coupling protein (*traG*), another hypothesis is that RcgA acts in a similar way to a coupling protein, being involved in the recruitment of a plasmid to the T4SS assembled in the membrane. Then, the absence of RcgA would not allow the interaction between the processed DNA and the Mpf. Another role could rely on the transport of a signal produced by the receptor strain. It has been recently described that the presence of a receptor strain increases the expression of conjugative genes in systems regulated by LuxR-like regulators (57). The fact that exogenous signals regulate CT has also been reported in pCF10 from Enterococcus faecalis through the transport of C peptide (49, 50). Thus, RcgA could transport a signal that is needed for CT from the receptor strain. Indeed, as RcgA/RcgR are encoded in an operon structure, it could be hypothesized that RcgR inactivates the signal transported by RcgA, which is needed for CT. In this way, both genes would create a fine tuning of CT in the presence of the receptor strain. Hence, the data presented here shows evidence of new actors that expand the knowledge of conjugative plasmid regulation. Future efforts will be devoted to elucidating the complete molecular system, thus allowing for a better understanding of how plasmids are disseminated between soil microorganisms.

## MATERIALS AND METHODS

### Bacterial strains and plasmids.

The strains and plasmids used in this work are listed in Table S4 (available at http://sedici.unlp.edu.ar/handle/10915/140513). Escherichia coli was grown on LB medium (58) at 37°C. *Rhizobium*, *Sinorhizobium*, *Agrobacterium* and *Shinella* strains were grown on TY (59) at 28°C. For solid media, 15 g of agar per liter of medium were added. The final concentrations of antibiotics used were (in μg mL^−1^): gentamicin (Gm) 10 and kanamycin (Km) 25 for E. coli. For *Rhizobium*, *Sinorhizobium*, *Agrobacterium* and *Shinella*: streptomycin (Sm) 400, neomycin (Nm) 60, rifampicin (Rif) 100, and gentamicin (Gm) 30.

### Bacterial matings.

The bacterial matings were performed as described by R. Simon et al. (60). Briefly, overnight cultures were grown to stationary-phase. The donor and recipient strains were mixed in a 1:1 ratio, plated on TY plates, and incubated overnight at 28°C. The bacteria were resuspended in 1 mL of 10 mM MgSO_4_-0.01% Tween 40 (vol/vol). For quantitative conjugations, Agrobacterium tumefaciens UBAPF2 was used as the recipient strain. Serial dilutions were plated on selective TY medium supplemented with the corresponding antibiotics to quantify the number of donor, recipient, and transconjugant cells. The conjugation frequencies were calculated as the ratio of transconjugants per donor cell. In each case, plasmid profiles were done by Eckhardt-gels (61), as modified by Hynes & McGregor (62), to corroborate the desired plasmid transfer.

### RNA extraction and synthesis of cDNA.

For RNA isolation, *R. favelukesii* LPU83 was cultivated in TY media. Then, RNAprotect (Qiagen, Hilden, Germany) reagent was added, the cells were harvested, and the pellet was frozen in liquid nitrogen. The commercial RNeasy R Protect Bacteria Mini Kit (Qiagen, Hilden, Germany) was used for the RNA extraction, and the DNA was removed with DNase I (Qiagen, Hilden, Germany).

The total RNA was retrotranscribed to cDNA using a Moloney murine leukemia virus (MMLV) reverse transcriptase (Thermo Fisher Scientific, Buenos Aires, Argentina), using random hexamers as primers.

### DNA manipulation, genetic constructs, and mutagenesis.

The total DNA and plasmid preparations, restriction-enzyme analysis, cloning procedures, and E. coli transformation were performed according to previously established techniques (63).

PCR amplification was carried out with recombinant *Taq* DNA polymerase or with *Phusion* DNA polymerase as specified by the manufacturers. The primers used in this study are listed in Table S5 (available at http://sedici.unlp.edu.ar/handle/10915/140513).

### Plasmid constructions for mutagenesis and the generation of deletional mutants.

For the construction of the vector to generate strain *R. favelukesii* LPU83-13 ΔLPU83a_0145, a C-terminal fragment of LPU83a_0145m was amplified with *Taq* polymerase and primers *145-Cter-Xba/145-Cter-Hind* (210 bp) and then cloned into pGem-T easy (Invitrogen), which yielded pGem::145Cter. Then, an N-terminal fragment of LPU83a_0145 was amplified with *Taq* polymerase and primers *145-Nter-Sal/145-Nter-Hind* (257 bp) and then cloned into the SmaI site of pK18mob, yielding pK18::145Nter. The pK18::145Nter was digested with SalI and HindIII, and the released fragment was cloned into the SalI/HindIII sites of pGem::145Cter, yielding pGem::Δ145. Then, pGem::Δ145 was digested with SalI and XbaI, and the released fragment was cloned into the SalI/XbaI sites of pJQ200KS, yielding pJQ200::Δ145 (5793 bp).

For the construction of the vector to generate strain *R. favelukesii* LPU83-13 ΔLPU83a_0146, two fragments of LPU83a_0146 were amplified with *Phusion* polymerase and primers *146-Cter-Xba/146-Cter-Bam* (199 bp) and *146-Nter-Sal/146-Nter-Bam* (201 bp). Both fragments were cloned into the SmaI site of pK18mob, yielding pK18::146Cter and pK18::146Nter, respectively. The pK18::146Nter was digested with SalI and BamHI, and the released fragment was cloned into the SalI/BamHI sites of pJQ200KS, yielding pJQ200::146Nter. Then, the pK18::146Cter was digested with BamHI and XbaI, and the released fragment was cloned into pJQ200::146Nter, yielding pJQ200::Δ146 (5722 bp).

For the construction of the vector to generate strain *R. favelukesii* LPU83-13 ΔLPU83a_0148, two fragments of LPU83a_0148 were amplified with *Phusion* polymerase and primers *148-Cter-Xba/148-Cter-Bam* (241 bp) and *148-Nter-Sal/148-Nter-Bam* (249 bp). Both fragments were cloned into the SmaI site of pK18mob, yielding pK18::148Cter and pK18::148Nter, respectively. The pK18::148Nter was digested with SalI and BamHI, and the released fragment was cloned into the SalI/BamHI sites of pJQ200KS, yielding pJQ200::148Nter. Then, the pK18::148Cter was digested with BamHI and XbaI, and the released fragment was cloned into pJQ200::148Nter, yielding pJQ200::Δ148 (5812 bp).

For the construction of the vector to generate strain *R. favelukesii* LPU83-13 ΔLPU83a_0148 ΔLPU83a_0146, pK18::146Cter was digested with BamHI and XbaI, and the released fragment was cloned into the BamHI/XbaI sites of pJQ200::148Nter, yielding pJQ200::Δ148Δ146 (5770 bp).

The constructed vectors were introduced by conjugation into *R. favelukesii* LPU83-13. Double recombinants were selected in TY saccharose 10% wt/vol as Nm^R^, and Gm^S^. To corroborate the deletions, PCRs were carried out with primers *145-l-out and 145-r-out* for deletion in LPU83a_0145 (581 bp), *146-l-out* and *146-r-out* for deletion in LPU83a_0146 (657 bp), *PCR3-r* and *148-r-out* for deletion in LPU83a_0148 (639 bp), and *146-l-out* and *148-r-out* for deletion in LPU83a_0146 and LPU83a_0148 (606 bp).

### Plasmid constructions for complementation assays and generation of complemented strains.

The vector for the LPU83a_0145 complementation assay was constructed as follows. A fragment containing the full LPU83a_0145 gene was amplified (757 bp) with *Phusion* polymerase and primers *145-l-Xba*/*145-r-Kpn*. The fragment was cloned into the SmaI site of pK18mob. The resulting vector was digested with KpnI/XbaI, and the released fragment was cloned into the KpnI/XbaI sites of pBBR1MCS-5, yielding pBBR::0145 (5,453 bp).

The vector for the *rcgR* complementation assay was constructed as follows. A fragment containing the full *rcgR* gene was amplified (1,079 bp) with *Phusion* polymerase and primers *146-l-Xba/146-r-Kpn*. The fragment was cloned into the SmaI site of pK18mob. The resulting vector was digested with KpnI/XbaI, and the released fragment was cloned into the KpnI/XbaI sites of pBBR1MCS-5, yielding pBBR::*rcgR* (5,775 bp).

The vector for the *rcgA* complementation assay was constructed as follows. A fragment containing the full *rcgA* gene was amplified (1,831 bp) with *Phusion* polymerase and primers *148-l-Xba/148-r-Kpn*. The fragment was cloned into the SmaI site of pK18mob. The resulting vector was digested with KpnI/XbaI, and the released fragment was cloned into the KpnI/XbaI sites of pBBR1MCS-5, yielding pBBR::*rcgA* (6,527 bp).

The vectors for the *rcgR* heterologous complementation assays were constructed as follows. A fragment containing the full *rcgR* homologous gene present in S. meliloti LPU88 (1,234 bp) or *Shinella* sp. DD12 (1,162 bp) was amplified with *Phusion* polymerase and primers *146-Cter-88/146-Nter-88* or *146-Cter-Sh/146-Nter-Sh*, respectively. The fragments were cloned into the SmaI site of pK18mob. The resulting vectors were digested with KpnI/XbaI, and the released fragments were cloned into the KpnI/XbaI sites of pBBR1MCS-5, yielding pBBR::*rcgR*-LPU88 (6,002 bp) or pBBR::*rcgR*-Shinella, respectively (6,034 bp).

The vectors for the *rcgA* heterologous complementation assays were constructed as follows. A fragment containing the full *rcgA* homologous gene present in S. meliloti LPU88 (1,885 bp) or *Shinella* sp. DD12 (1,804 bp) was amplified with *Phusion* polymerase and primers *148-Cter-88/148-Nter-88* or *148-Cter-Sh/148-Nter-Sh*, respectively. The fragments were cloned into the SmaI site of pK18mob. The resulting vectors were digested with KpnI/XbaI, and the released fragments were cloned into the KpnI*/*XbaI sites of pBBR1MCS-5, yielding pBBR::*rcgA*-LPU88 (6,653 bp) or pBBR::*rcgA*-Shinella, respectively (6,509 bp).

For the generation of complemented strains, the constructed vectors were transformed into E. coli S17-1, and then biparental matings to the respective strains were carried out. The transconjugants were selected as Nm^r^ and Gm^r^

### Plasmid construction and the generation of Gm resistant strains.

For the construction of the strain S. meliloti LPU88 Gm, an intergenic region between *rptA* and ORF4 (accession number JQ753316.1) from the plasmid pLPU88a was amplified with *Phusion* polymerase and primers *Gm-88a-left/Gm-88a-right*. The obtained fragment (239 bp) was cloned into the SmaI site of pG18mob2, yielding pG18mob2::88a (3,120 bp). To introduce pG18mob2::88a into S. meliloti LPU88, the pG18mob2::88a was first transformed in E. coli S17-1, and then biparental matings were carried out. The simple recombinants were selected as Sm^r^ and Gm^r^. To corroborate the insertion, PCR was carried out with primers *88a-check/m13rv-40* (772 bp).

For the construction of the strain *Shinella* sp. DD12 Gm, an intergenic region between genes SHLA_RS09770 and SHLA_RS09775 from the plasmid pDD12c was amplified with *Phusion* polymerase and primers *Gm-Sh-left/Gm-Sh-right*. The obtained fragment (220 bp) was cloned into the SmaI site of pG18mob2, yielding pG18mob2::Sh (3,101 bp). To introduce pG18mob2::Sh into *S.* sp. DD12, the pG18mob2::Sh was first transformed in E. coli S17-1, and then biparental matings were carried out. The simple recombinants were selected as Sm^r^ and Gm^r^. To corroborate the insertion, PCR was carried out with primers *Sh-check/m13rv-40* (705 bp).

### Plasmid construction for *traR*::GFP fusion and the generation of the strains carrying the construction.

For the construction of pG18mob2::*traR*::GFP, the full *traR* gene from pLPU83a was amplified with *Phusion* polymerase and primers *traR-Nter/traR-Cter*. The obtained fragment (788 bp) was cloned into the SmaI site of pG18mob2, yielding pG18mob2::*traR* (3,669 bp). To introduce GFP in this plasmid, the full GFP gene was released from pMP6 with EcoRI and cloned into the EcoRI site of pG18mob2::*traR*, yielding pG18mob2::*traR*::GFP (4,446 bp). The orientation of GFP was confirmed by the restriction pattern generated by KpnI. To introduce pG18mob2::*traR*::GFP into *R. favelukesii* LPU83-13 and its derivative mutants, the pG18mob2::*traR*::GFP was first transformed in E. coli S17-1, and then biparental matings were carried out. The simple recombinants were selected as Nm^r^ and Gm^r^. To corroborate the insertion, PCR was carried out with primers *traR-out*/*m13rv-40* (1,890 bp). For the assays in A. tumefaciens UBAPF2, pLPU83a-13 and its derivative mutants containing the *traR*::GFP fusion were transferred to A. tumefaciens UBAPF2 by conjugation. The transconjugants were selected as Gm^r^, Nm^r^, and Rif^r^.

### Bioinformatics 3-D structure predictions.

For structural predictions, a simplified version of AlphaFold v2.1.0 (33) was used. PDB files were visualized with PyMOL 2.5 (The PyMOL Molecular Graphics System, Version 2.5, Schrödinger, LLC). The structural comparisons were done by using the Dali server (34) against the PDB database. The analyses of the transmembrane domains were done with TMHMM-2.0 (31, 32).

### Taxonomic analysis.

A BLASTP analysis on the NCBI server was used to obtain the homologs. For the metagenomic proteins, we used the env nr database from NCBI. For the homologs in isolated bacteria, we used the nonredundant protein sequences (nr) database from NCBI. The data are actualized to February 2, 2022. Genetic regions were examined in the neighborhoods of the selected genes using the nucleotide visualizer tool from NCBI. As criteria for protein homology, we used the parameters of greater than 70% coverage and greater than 30% identity (64).

### Phylogenetic analysis.

For the construction of the RcgR (LPU83a_0146) and RcgA (LPU83a_0148) phylogenetic trees, the proteins were aligned with the module of MUSCLE implemented in MEGA5 (65). The models of protein evolution for our sequences were selected with Prottest2.4 (66). The best model was JTT + I + G + F for both trees. Maximum likelihood (ML) trees were inferred under the selected model using PhyML v3.1 (67). The robustness of the ML topologies was evaluated using a Shimodaira-Hasegawa-like test for branches, implemented in PhyML v3.1. We employed the best of the nearest-neighbor interchange and shortest path routing algorithms to search the topologies of the trees and used 100 random trees as the initial trees.

The accession numbers for the proteins selected for the LPU83a_0146 and LPU83a_0148 phylogenies are listed in Table S2 (available at http://sedici.unlp.edu.ar/handle/10915/140513).

### GFP transcriptional fusions and fluorescence assays.

To evaluate *traR* promoter activity, we designed a strategy based on a transcriptional fusion between the GFP protein and *traR*, considering that an increment in *traR* transcription would lead to an increment in GFP expression. Details of the construction of pG18mob:*traR*::GFP are listed before.

Once the strains carrying the construction were obtained, fluorescence was measured as follows: cultures were grown on TY liquid media until stationary-phase. After an OD_600_ measurement, a bacterial pellet normalized to OD = 1 was resuspended in 200 μL of NaCl 0.9% (wt/vol). Fluorescence was measured in a DeNovix QFX fluorometer. The values are expressed in arbitrary units (AU).

### Proteomics analyses.

For the protein isolation, 1.5 mL of cell cultures were centrifuged at 13,000 rpm for 5 min, and pellets were frozen in N_2_. Pellets were resuspended in 100 μL of 100 mM Ambic (ammonium bicarbonate [Honeywell Fluka]). Next, 100 μL of the organic solvent trifluoroethanol (TFE, Fluka biochemika) and 5 μL of 20 nM DTT were added. The tubes were mixed by inversion and incubated at 60°C for 60 min. In a second step, 20 μL of 200 mM iodoacetamid (IAA) was added, and the tubes were then placed in the dark at room temperature for 90 min. Then, 5 μL of 20 nM DTT were added again, and the tubes were then incubated at room temperature for 60 min. Half of the mixture was saved at –80°C as a backup. Then, 437 μL of 100 mM ammonium bicarbonate and 437 μL of bidistilled water were added to the remaining sample. In the next step, 10 μL trypsin gold (1 μg μL^−1^) (Mass Spectrometry Grade, Promega, WI, USA) were added, and the solution was incubated at 37°C overnight.

The protein digestion was purified the next day with Sep Pak C18 cartridges (Waters Corporation, Milford, MA, USA). The Sep Pak C18 cartridges were rinsed with 1 mL of solution B (65% Acetonitril, 35% bidistilled water; 0.1% TFA). The column was equilibrated with 1 mL of solution A (98% Acetonitril, 2% bidistilled water; 0.1% TFA). Next, the digest mixed with 1 mL of solution A was added and run through the column slowly. Subsequently, the cartridges were washed with 1 mL of solution A. The proteome was eluted with 100 μL of solution B in low protein binding collection tubes and then dried in a vacufuge concentrator (Eppendorf, Hamburg, Germany). Next, the dried peptide mixture was resuspended in 15 μL of solution A and quantified with a Nano-Drop 2000/2000c (Thermo Fisher, MA, USA).

LC-MS/MS measurements were carried out using a QExactive mass spectrometer (Thermo Fisher Scientific, Waltham, MA, USA) that was online coupled to an LC system. The peptides were separated on a 25 cm steel column Acclaim PepMap 100 C18-LC-column with a particle size of 2 μm and a diameter of 75 μm (Thermo Fisher, MA, USA). Identification and a label-free quantification (LFQ) analysis were performed using the software MaxQuant with the default settings and a false discovery rate of p_adj_ < 0.05 (68). In total, 253,381 MS/MS spectra were recorded, resulting in 18,114 identified peptide sequences corresponding to 2,375 proteins.

The statistical analysis of the LFQ data obtained from MaxQuant was performed with Perseus 1.6.10.43 (69). 2,078 proteins remained when at least 2 unique peptides were used as the threshold for identification. Among these, the normalized LFQ intensities became null in all of the samples for 440 of them. Thus, the final protein list contains 1,638 proteins.

### Data availability.

The mass spectrometry proteomics data have been deposited to the ProteomeXchange Consortium (70) via the PRIDE partner repository (71) with the data set identifier PXD033583.
